# Supplementary material for: A Multisample Approach in Forensic Phenotyping of Chronological Old Skeletal Remains Using Massive Parallel Sequencing (MPS) Technology
Source: Genes (Basel). 2023 Jul 14;14(7):1449. doi: 10.3390/genes14071449 (PMC10379588; doi:10.3390/genes14071449)
Supplement: Supplementary file 1 [file genes-14-01449-s001.zip › S3.pdf]

Table S3: Coverage values for HIrisPlex SNP markers and their corresponding genes for 24 bone samples from 8 skeletons excavated from the Huda Jama Mass Grave. Threshold for >100 pg DNA input is shown in third column.

Gene	SNP marker	Treshold	Coverage values							
			Sample 1	Sample 2	Sample 3	Sample 4	Sample 5	Sample 6	Sample 7	Sample 8
MC1R	rs 11547464	249	37542	41059	81243	13319	45893	79117	78973	98757
MC1R	rs 885479	246	37259	40686	80539	13125	45149	78551	78813	99982
MC1R	rs1805008	249	37549	41203	81211	13307	45851	79153	79406	99950
MC1R	rs 1805005	308	14914	10618	16270	7306	12353	21692	30444	16761
MC1R	rs 1805006	318	14889	10666	16330	7363	12413	21817	30533	16824
MC1R	rs 1805007	249	37570	41255	81271	13334	45948	79226	79095	98836
TUBB3	rs 1805009	266	32792	32930	73158	15386	240838	41949	66347	72780
MC1R	rs 201326893	249	37605	41277	80612	13349	45976	79267	79539	99986
MC1R	rs 2228479	320	14818	10445	15700	7190	12101	21285	30312	16473
MC1R	rs 1110400	248	37568	41254	81288	13340	45934	79238	79479	99572
SLC45A2	rs 28777	307	64987	52916	99704	38338	64648	99716	88374	69083
SLC45A2	rs 16891982	179	8727	3265	4478	3155	12229	13100	12673	14483
KITLG	rs 12821256	215	14866	5125	8618	3982	10738	14518	17591	40604
LOC105374875	rs 4959270	201	15952	7823	11967	4755	12227	16509	17889	23764
IRF4	rs 12203592	206	34991	14310	33389	13371	40297	66578	52783	70818
TYR	rs 1042602	210	38507	22419	41412	14690	47402	49586	37226	52033
OCA2	rs 1800407	288	40494	20478	41174	15373	32248	65435	68649	57026
SLC24SA4	rs 2402130	286	37871	28081	46674	16852	33883	47031	32636	34740
HERC2	rs 12913832	391	53095	28765	52825	18528	64089	72979	76645	98973
PIGU	rs 2378249	182	51181	44443	62098	25151	57484	72870	53424	41925
LOC105370627	rs 12896399	284	41970	28540	48012	20220	40538	64760	41825	59710
TYR	rs 1393350	189	66371	38460	78699	24563	45513	97140	50002	63386
TYRP1	rs 683	254	26340	12059	23333	8891	28938	38930	27093	27123

Gene	Marker	Treshold	Coverage values (continued)							
			Sample 9	Sample 10	Sample 11	Sample 12	Sample 13	Sample 14	Sample 15	Sample 16
MC1R	rs 11547464	249	75444	18000	20004	43055	30413	19615	31708	38082
MC1R	rs 885479	246	74998	17827	19824	42843	30153	19459	31511	37849
MC1R	rs1805008	249	75370	17989	19992	43190	30391	19632	31453	38100
MC1R	rs 1805005	308	29092	7580	9420	10226	8210	8203	7895	20868
MC1R	rs 1805006	318	29122	7616	9449	10274	8197	8228	7923	20874
MC1R	rs 1805007	249	75021	18015	41244	43268	30430	19657	31727	38132
TUBB3	rs 1805009	266	52460	15769	34707	31172	31321	22476	33181	35975
MC1R	rs 201326893	249	75864	18028	20021	43272	30178	19663	31751	27871
MC1R	rs 2228479	320	28985	7532	9178	10096	8189	8129	7832	20603
MC1R	rs 1110400	248	76177	18018	20020	43244	30444	19645	31720	38105
SLC45A2	rs 28777	307	47671	27426	68458	82661	57760	43243	74540	80139
SLC45A2	rs 16891982	179	4185	7352	13285	6771	15160	8017	12002	15427
KITLG	rs 12821256	215	9151	9574	15968	10913	21337	14382	21425	21795
LOC10537 4875	rs 4959270	201	10612	6984	14205	7329	11677	9892	10930	18470
IRF4	rs 12203592	206	20858	19023	48941	43764	62682	44282	53318	47237
TYR	rs 1042602	210	20542	26094	38503	31511	58819	35653	59102	51197
OCA2	rs 1800407	288	24464	22341	41244	38247	59238	44465	54383	45806
SLC24SA4	rs 2402130	286	29260	9501	27665	40254	24842	17354	20480	36120
HERC2	rs 12913832	391	31340	31735	67196	76494	90612	59558	86923	63485
PIGU	rs 2378249	182	46221	17052	37599	43898	38553	22103	36192	66935
LOC10537 0627	rs 12896399	284	23998	15942	35671	40544	39744	28013	40642	46811
TYR	rs 1393350	189	37634	21259	47509	51420	50969	27794	49743	65476
TYRP1	rs 683	254	12872	19687	29425	23520	32910	22737	26397	40830

Gene	Marker	Treshold	Coverage values (continued)							
			Sample 17	Sample 18	Sample 19	Sample 20	Sample 21	Sample 22	Sample 23	Sample 24
MC1R	rs 11547464	249	47311	42115	62899	17489	27004	42366	36730	37351
MC1R	rs 885479	246	46742	41767	62244	17315	26661	42023	36251	37135

MC1R	rs1805008	249	47277	42137	62883	17485	26949	42354	36702	37319
MC1R	rs 1805005	308	20039	19044	33195	11514	15280	28834	20377	30061
MC1R	rs 1805006	318	20083	19116	33360	11576	15293	28922	20494	30184
MC1R	rs 1805007	249	47331	42141	62941	17492	27052	42350	36751	37303
TUBB3	rs 1805009	266	45073	33665	55744	13404	19623	33791	33620	33464
MC1R	rs 201326893	249	47380	42161	62990	17521	27070	42419	36772	37389
MC1R	rs 2228479	320	19855	18913	32958	11444	15185	28586	20268	29801
MC1R	rs 1110400	248	47324	42130	62936	17513	27036	42369	36698	37337
SLC45A2	rs 28777	307	68501	74009	99744	19914	31884	63502	53167	57143
SLC45A2	rs 16891982	179	13994	11684	18296	2324	4387	12630	10389	17147
KITLG	rs 12821256	215	15712	15581	22356	3309	4808	13398	13063	17952
LOC105374875	rs 4959270	201	17554	18085	24708	5363	9182	16328	16058	21986
IRF4	rs 12203592	206	43006	36657	57875	8186	11968	34638	30174	36788
TYR	rs 1042602	210	42659	44612	63590	8024	13869	31971	33889	35554
OCA2	rs 1800407	288	39108	34247	54601	7850	11152	24678	24269	27355
SLC24SA4	rs 2402130	286	29848	35213	57712	10209	14867	29857	34949	27779
HERC2	rs 12913832	391	64546	56675	86769	11936	18047	49508	46628	42708
PIGU	rs 2378249	182	50393	51533	68362	17990	28325	42893	42048	50941
LOC105370627	rs 12896399	284	42923	46031	65131	10747	19302	41464	38981	41025
TYR	rs 1393350	189	55846	56825	85102	17129	29529	56260	48725	63220
TYRP1	rs 683	254	30067	32785	49605	7745	12421	28565	25490	39943

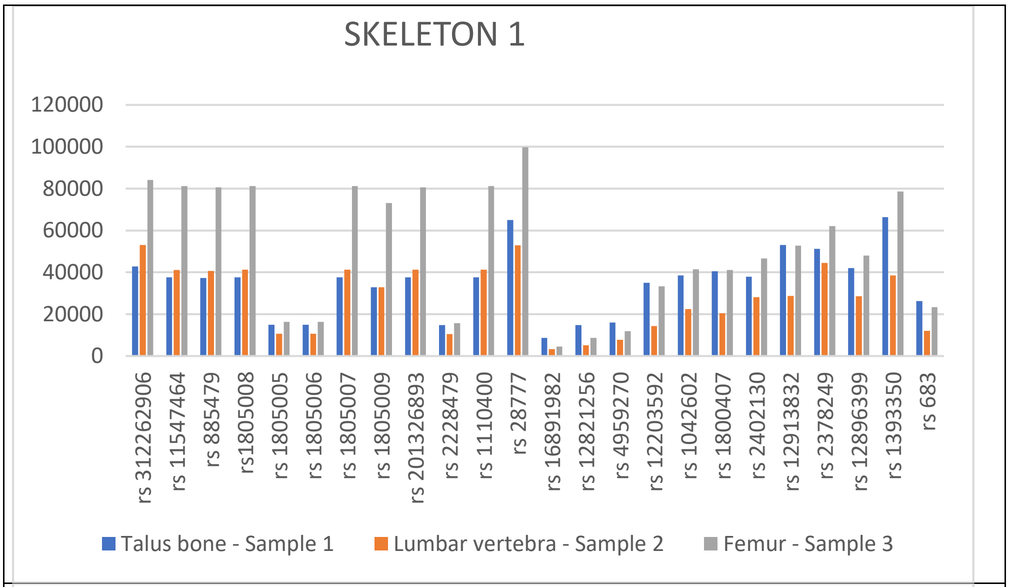

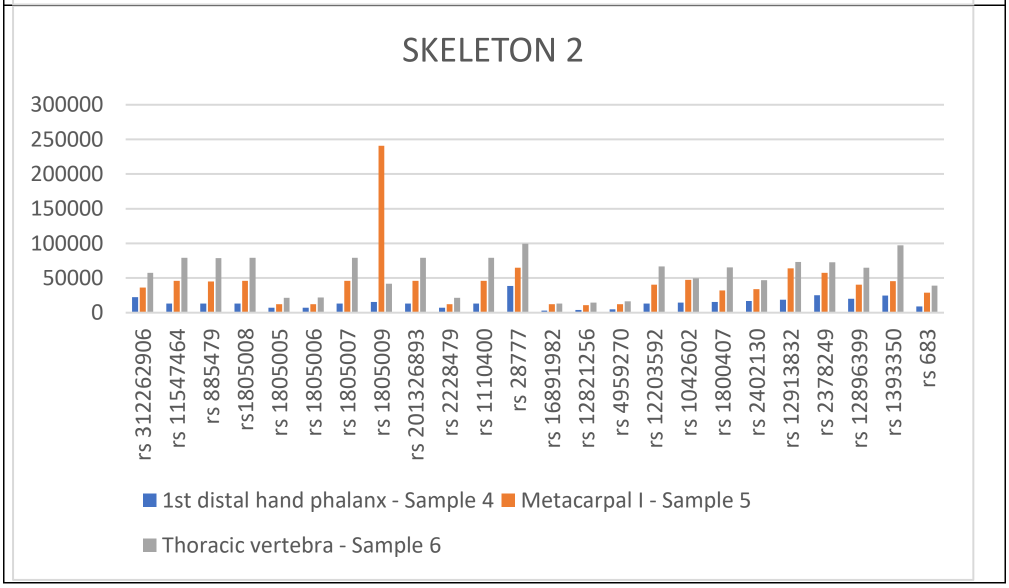

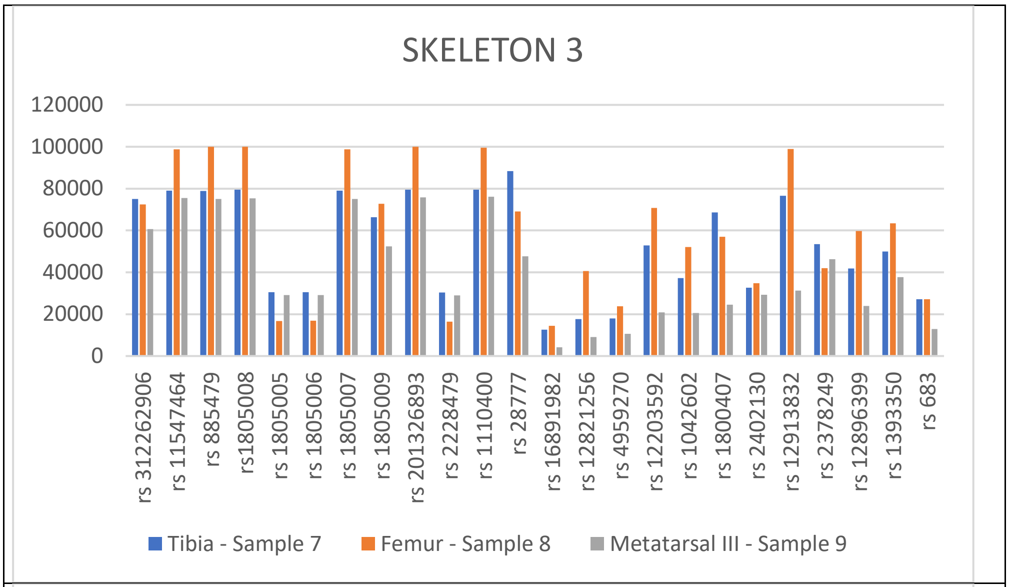
SKELETON 3
120000
100000
80000
60000
40000
20000
0
rs 312262906
rs 11547464
rs 885479
rs1805008
rs 1805005
rs 1805006
rs 1805007
rs 1805009
rs 201326893
rs 2228479
rs 1110400
rs 28777
rs 16891982
rs 12821256
rs 4959270
rs 12203592
rs 1042602
rs 1800407
rs 2402130
rs 12913832
rs 2378249
rs 12896399
rs 1393350
rs 683
Tibia - Sample 7
Femur - Sample 8
Metatarsal III - Sample 9

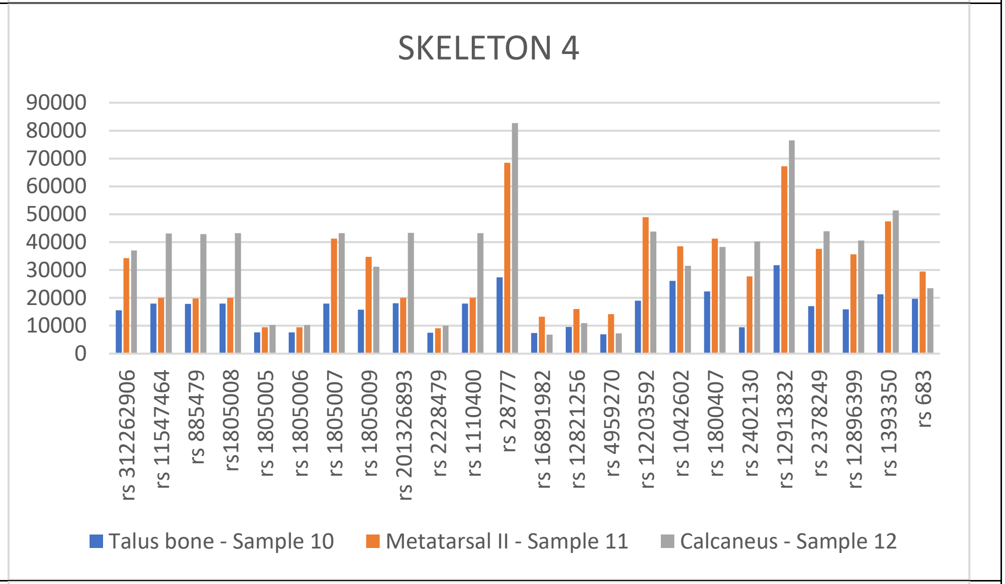
SKELETON 4
90000
80000
70000
60000
50000
40000
30000
20000
10000
0
rs 312262906
rs 11547464
rs 885479
rs1805008
rs 1805005
rs 1805006
rs 1805007
rs 1805009
rs 201326893
rs 2228479
rs 1110400
rs 28777
rs 16891982
rs 12821256
rs 4959270
rs 12203592
rs 1042602
rs 1800407
rs 2402130
rs 12913832
rs 2378249
rs 12896399
rs 1393350
rs 683
Talus bone - Sample 10
Metatarsal II - Sample 11
Calcaneus - Sample 12

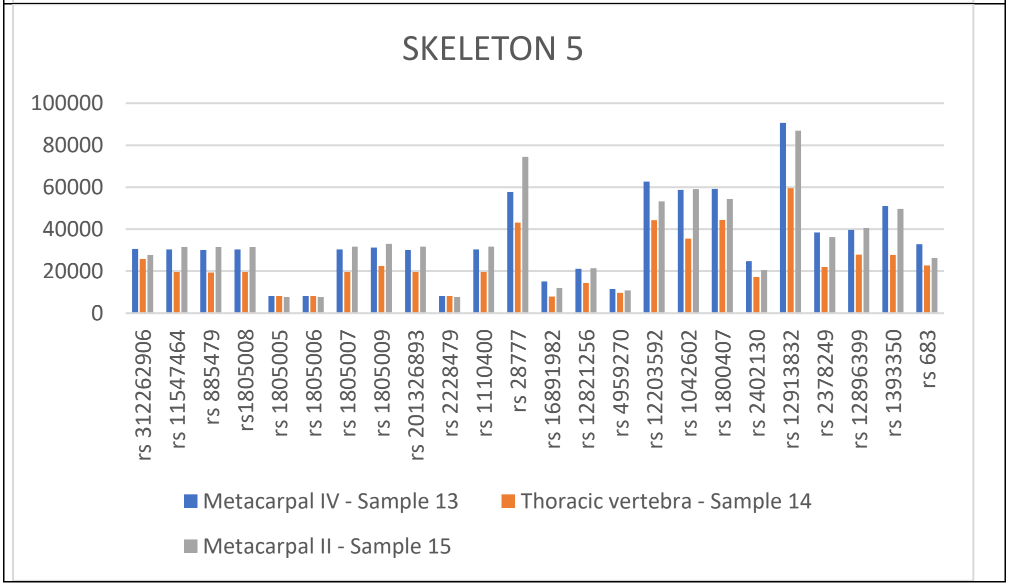
SKELETON 5
100000
80000
60000
40000
20000
0
rs 312262906
rs 11547464
rs 885479
rs1805008
rs 1805005
rs 1805006
rs 1805007
rs 1805009
rs 201326893
rs 2228479
rs 1110400
rs 28777
rs 16891982
rs 12821256
rs 4959270
rs 12203592
rs 1042602
rs 1800407
rs 2402130
rs 12913832
rs 2378249
rs 12896399
rs 1393350
rs 683
Metacarpal IV - Sample 13
Thoracic vertebra - Sample 14
Metacarpal II - Sample 15

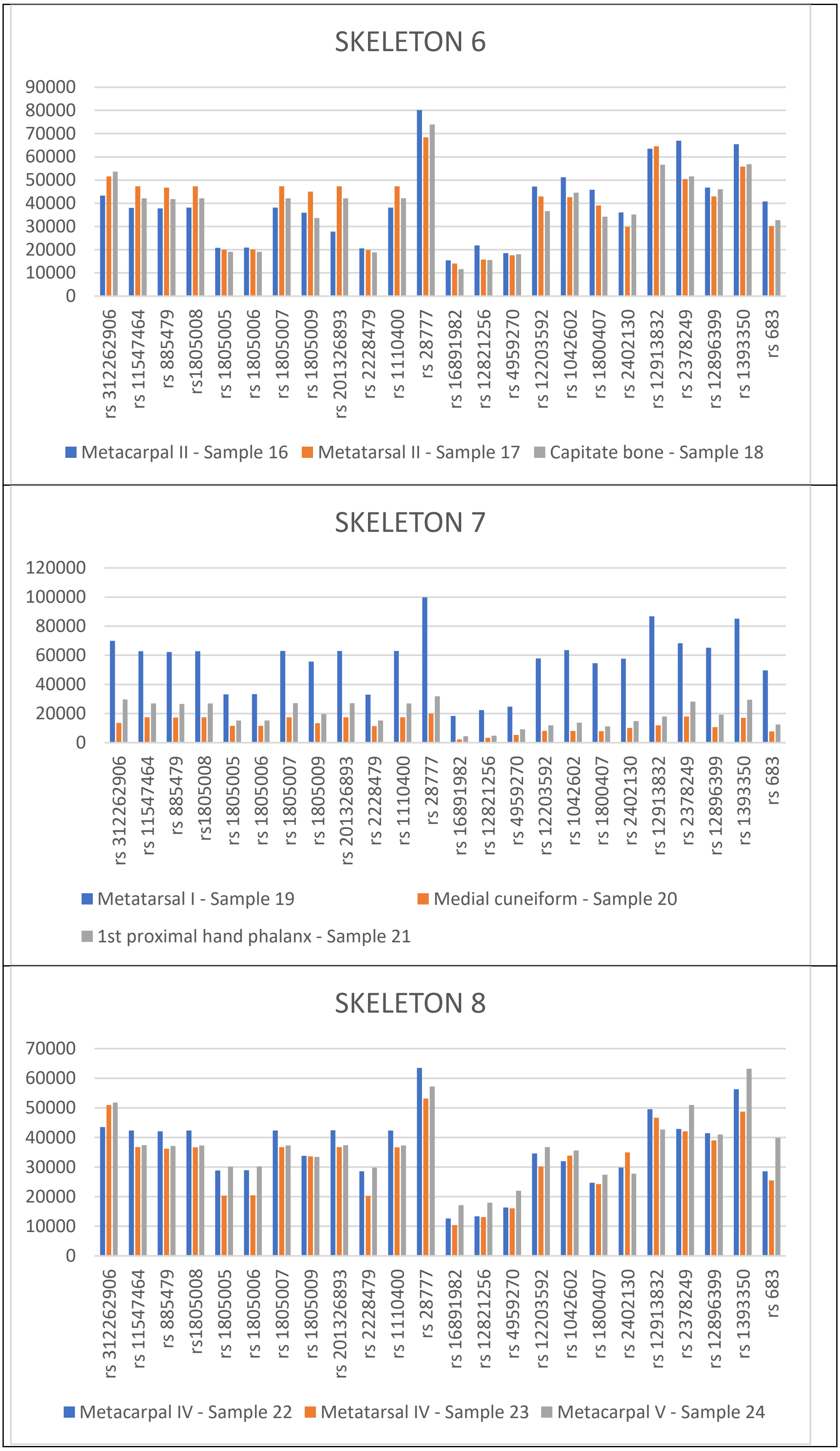

Figure S2: coverage values for HIrisPlex SNPs for three different skeletal elements per each skeleton analyzed from the Huda Jama Mass Grave.
