# Supplementary material for: A Multisample Approach in Forensic Phenotyping of Chronological Old Skeletal Remains Using Massive Parallel Sequencing (MPS) Technology
Source: Genes (Basel). 2023 Jul 14;14(7):1449. doi: 10.3390/genes14071449 (PMC10379588; doi:10.3390/genes14071449)
Supplement: Supplementary file 1 [file genes-14-01449-s001.zip › S4.pdf]

Table S4: Average coverage values for HIrisPlex SNP markers for eight skeletons excavated from the Huda Jama Mass Grave. Averages are calculated from three skeletal elements per skeleton.

MARKER	skeleton 1	skeleton 2	skeleton 3	skeleton 4	skeleton 5	skeleton 6	skeleton 7	skeleton 8
rs 312262906	60032	38714	69379	28932	28144	49503	37764	48770
rs 11547464	53281	46110	84391	27020	27245	42503	35797	38816
rs 885479	52828	45608	84598	26831	27041	42119	35407	38470
rs1805008	53321	46104	84909	27057	27159	42505	35772	38792
rs 1805005	13934	13784	25432	9075	8103	19984	19996	26424
rs 1805006	13962	13864	25493	9113	8116	20024	20076	26533
rs 1805007	53365	46169	84317	34176	27271	42535	35828	38801
rs 1805009	46293	99391	63862	27216	28993	38238	29590	33625
rs 201326893	53165	46197	85130	27107	27197	39137	35860	38860
rs 2228479	13654	13525	25257	8935	8050	19790	19862	26218
rs 1110400	53370	46171	85076	27094	27270	42520	35828	38801
rs 28777	72536	67567	68376	59515	58514	74216	50514	57937
rs 16891982	5490	9495	10447	9136	11726	13702	8336	13389
rs 12821256	9536	9746	22449	12152	19048	17696	10158	14804
rs 4959270	11914	11164	17422	9506	10833	18036	13084	18124
rs 12203592	27563	40082	48153	37243	53427	42300	26010	33867
rs 1042602	34113	37226	36600	32036	51191	46156	28494	33805
rs 1800407	34049	37685	50046	33944	52695	39720	24534	25434
rs 2402130	37542	32589	32212	25807	20892	33727	27596	30862
rs 12913832	44895	51865	68986	58475	79031	61569	38917	46281
rs 2378249	52574	51835	47190	32850	32283	56287	38226	45294
rs 12896399	39507	41839	41844	30719	36133	45255	31727	40490
rs 1393350	61177	55739	50341	40063	42835	59382	43920	56068
rs 683	20577	25586	22363	24211	27348	34561	23257	31333

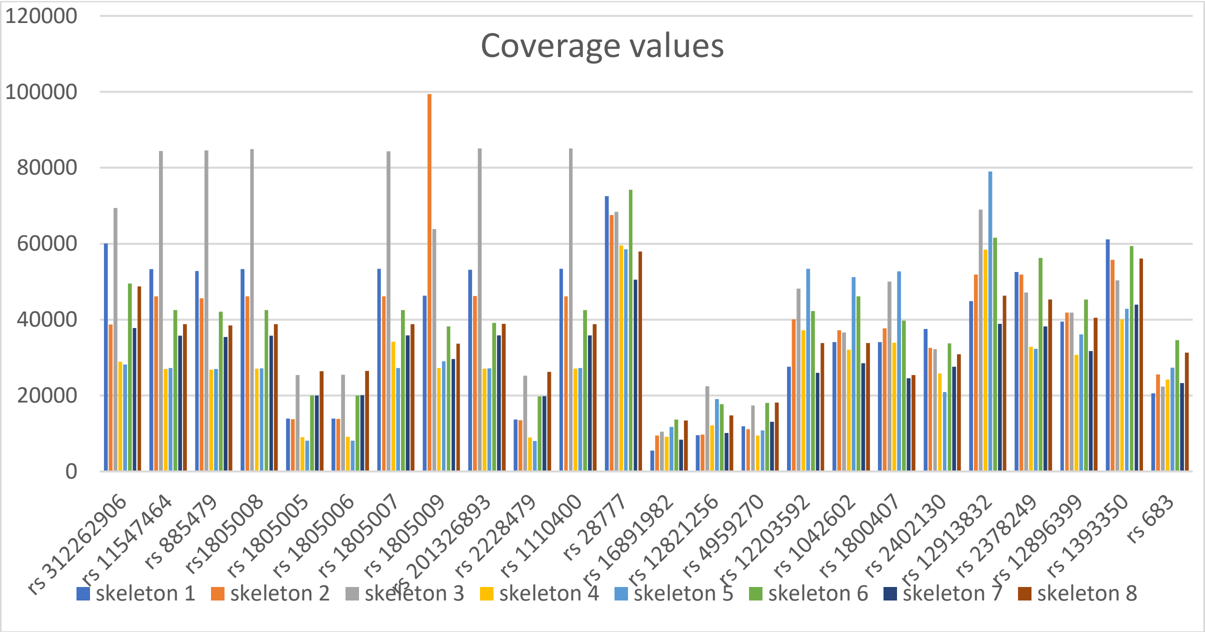

Figure S3: Average coverage values for HIrisPlex SNP markers for eight skeletons excavated from the Huda Jama Mass Grave. Averages are calculated from three skeletal elements per skeleton.
