# Supplementary material for: A Multisample Approach in Forensic Phenotyping of Chronological Old Skeletal Remains Using Massive Parallel Sequencing (MPS) Technology
Source: Genes (Basel). 2023 Jul 14;14(7):1449. doi: 10.3390/genes14071449 (PMC10379588; doi:10.3390/genes14071449)
Supplement: Supplementary file 1 [file genes-14-01449-s001.zip › S5.pdf]

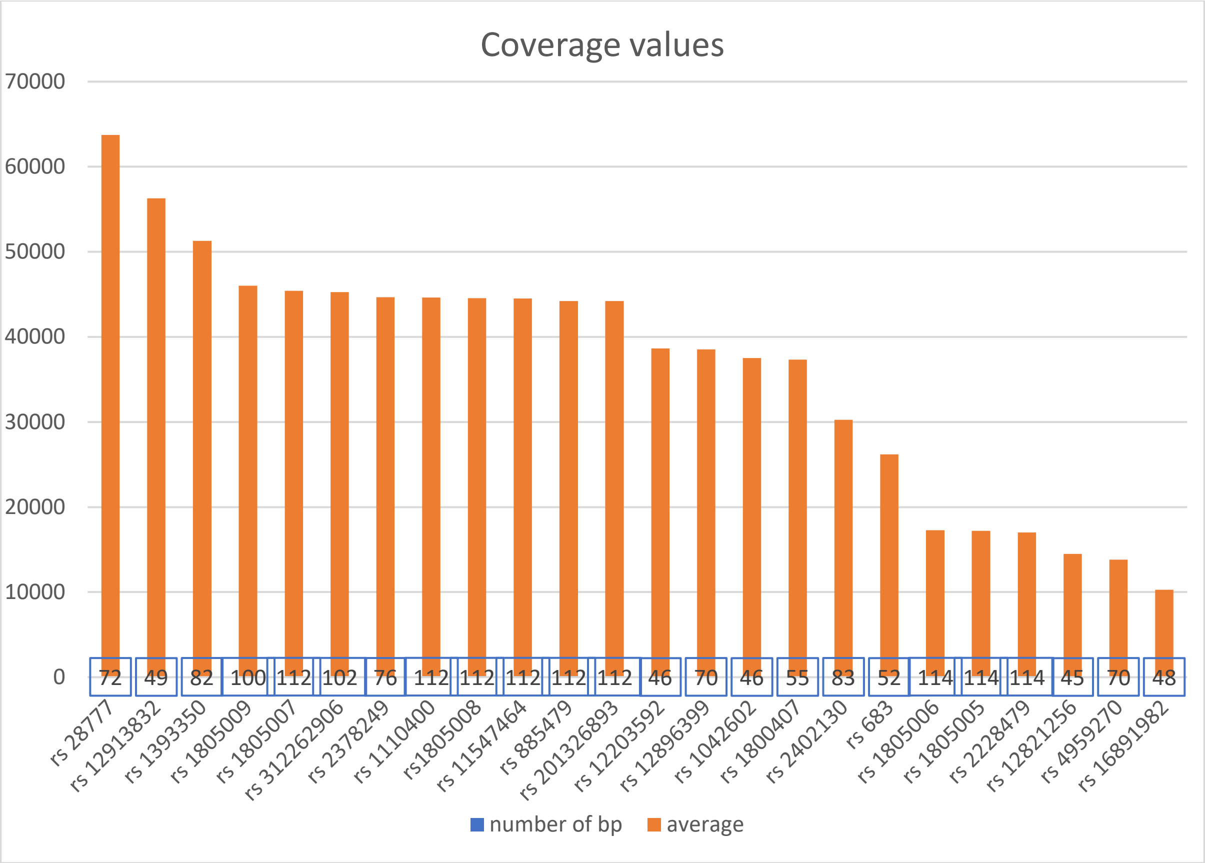

Figure S4: Average coverage values for HIrisPlex SNP markers for eight skeletons analyzed from the Huda Jama Mass Grave. Averages are calculated from 24 bones (three per each skeleton) analyzed per each SNP marker. Together with the name of the HIrisPlex SNP marker its length is shown in bp.
