# Supplementary material for: A Multisample Approach in Forensic Phenotyping of Chronological Old Skeletal Remains Using Massive Parallel Sequencing (MPS) Technology
Source: Genes (Basel). 2023 Jul 14;14(7):1449. doi: 10.3390/genes14071449 (PMC10379588; doi:10.3390/genes14071449)
Supplement: Supplementary file 1 [file genes-14-01449-s001.zip › S1.pdf]

Table S1: Characteristics (skeleton, sample number, skeletal type, extraction negative control - ENC), PowerQuant results (DNA quantity - Auto target, Deg target – both expressed in ng DNA in µl of extract , IPC shift and degradation index - DI), DNA quantity expressed in ng DNA per g of bone powder, PCR-MPS HIrisPlex template DNA input expressed in ng of DNA, and effectiveness of autosomal ESI 17 Fast STR typing - expressed as the number of successfully typed loci out of all loci present in amplification kit (16 STR loci and amelogenin).

Skeleton, sample no.	Skeletal type	DNA quantity-AUTO target (ng/µl)	DEG target (ng/µl)	IPC shift	Deg. Index (DI)	DNA quantity (ng/g bone)	PCR-MPS HIrisPlex template DNA input quantity (ng)	ESI 17 Fast (STR, amelogenin)
1-S 1	**Talus bone**	0.067	0.026	-0.05	2.6	6.7	1.00	17/17
1-S 2	Lumbar vertebra	0.017	0.005	-0.32	3.8	1.7	0.26	17/17
1-S 3	Femur	0.015	0.007	-0.43	2.2	1.5	0.23	17/17
2-S 4	1st distal hand phalanx	0.014	0.001	-0.85	16.0	1.4	0.21	17/17
2-S 5	Metacarpal I	0.013	0.002	-0.85	7.4	1.3	0.20	17/17
2-S 6	Thoracic vertebra	0.020	0.001	-0.82	16.0	2.0	0.30	17/17
3-S 7	**Tibia**	0.092	0.012	-0.49	7.6	9.2	1.00	17/17
3-S 8	Femur	0.060	0.011	-0.54	5.2	6.0	0.90	17/17
3-S 9	Metatarsal III	0.020	0.001	-0.27	16.3	2.0	0.30	17/17
4-S 10	Talus bone	0.039	0.004	-0.58	9.6	3.9	0.59	17/17
4-S 11	Metatarsal II	0.010	/	-0.16	undet	1.0	0.15	11/17
4-S 12	Calcaneus	0.017	0.001	-0.56	16.1	1.7	0.26	17/17
5-S 13	**Metacarpal IV**	0.087	0.003	-0.39	33.4	8.7	1.00	17/17
5-S 14	**Thoracic vertebra**	0.114	0.020	-0.4	5.8	11.4	1.00	17/17
5-S 15	**Metacarpal II**	0.105	0.003	-0.36	40.0	10.5	1.00	17/17
6-S 16	**Metacarpal II**	0.234	0.021	-0.46	11.2	23.4	1.00	17/17
6-S 17	**Metatarsal II**	0.482	0.041	-0.33	11.7	48.2	1.00	17/17
6-S 18	**Capitate bone**	0.279	0.047	-0.50	5.9	27.9	1.00	17/17
7-S 19	**Metatarsal I**	0.402	0.101	-0.04	4.0	40.2	1.00	17/17
7-S 20	**Medial cuneiform**	0.221	0.046	-0.19	4.9	22.1	1.00	17/17
7-S 21	**1st proximal hand phalanx**	0.292	0.080	-0.23	3.7	29.2	1.00	17/17
8-S 22	**Metacarpal IV**	0.374	0.039	-0.31	9.5	37.4	1.00	17/17
8-S 23	**Metatarsal IV**	0.164	0.010	-0.31	16.2	16.4	1.00	17/17
8-S 24	**Metacarpal V**	0.284	0.038	-0.42	7.5	28.4	1.00	17/17
ENC 1		/	/	-0.34	/	/		0/17
ENC 2		/	/	-0.14	/	/		0/17
ENC 3		/	/	-0.40	/	/		0/17
ENC 4		/	/	-0.36	/	/		0/17

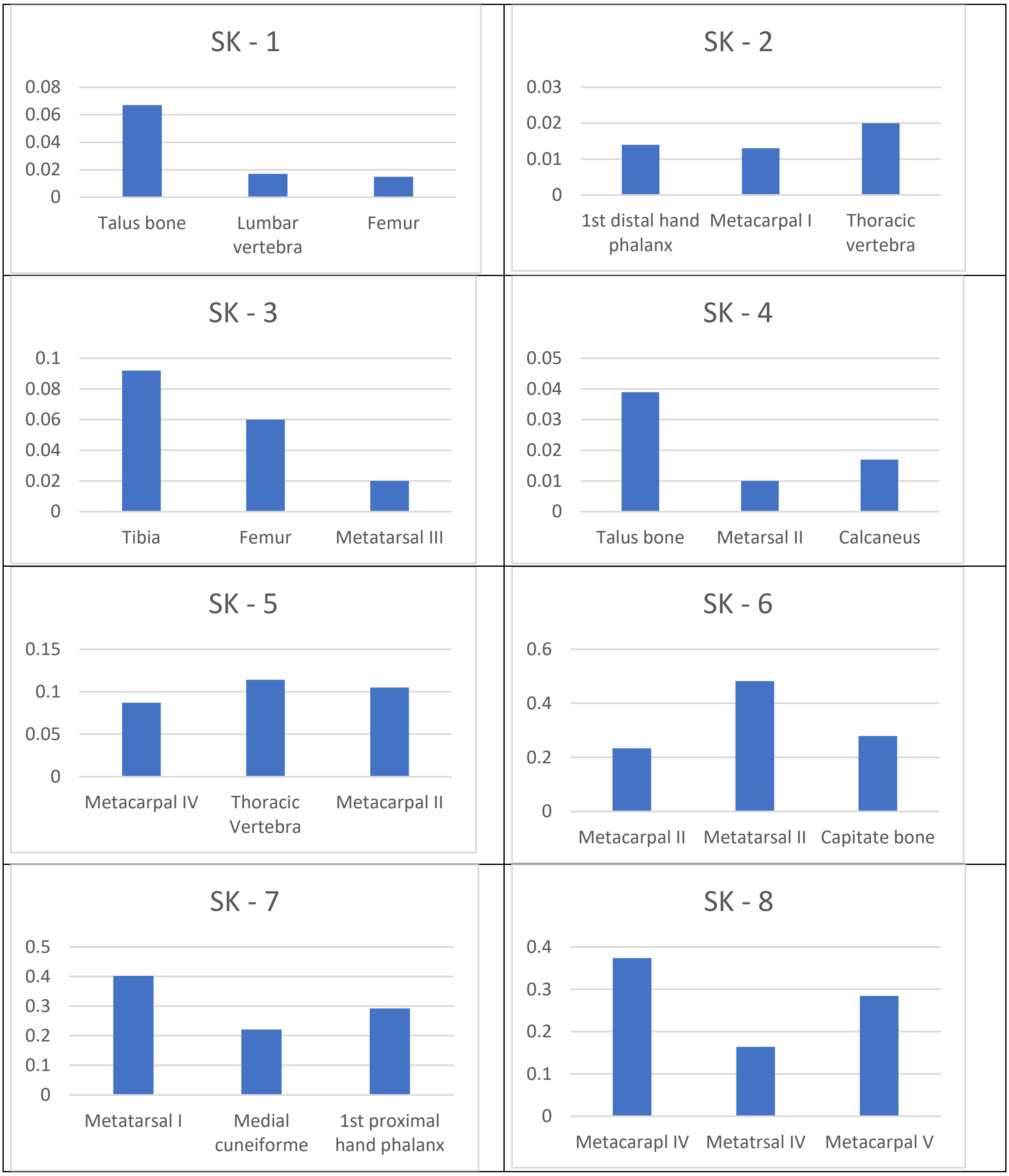

Figure S1: DNA quantity - PowerQuant Auto target (expressed in ng DNA/μL of extract) for three skeletal element types analyzed per each skeleton.
